# Hospital Expenditure for Microsurgical Peripheral Nerve Repair and the Broader Social Security Burden of Hand and Wrist Trauma in Brazil (2014-2024): An Ecological Analysis

**DOI:** 10.7759/cureus.114777

**Published:** 2026-08-19

**Authors:** Mariana Kelly Souto Menezes, Rudá Guimarães Rocha Justino, Daniel Torres Jacome

**Affiliations:** 1 Department of Medical Sciences, Hospital Universitário Antônio Pedro, Universidade Federal Fluminense, Niterói, Rio de Janeiro, BRA; 2 Department of Trauma and Orthopedics, Centro Universitário FACISA (UNIFACISA), Campina Grande, BRA

**Keywords:** cost analysis, epidemiology, hand injuries, peripheral nerves, social security

## Abstract

Objective: This study aimed to analyze the epidemiological profile and inflation-adjusted hospital expenditure associated with microsurgical peripheral nerve repairs performed in the Brazilian Unified Health System from 2014 to 2024 and, separately, to contextualize these findings through an ecological analysis of aggregated social security indicators related to traumatic hand, wrist, and related upper-limb injuries, without patient-level linkage between the databases.

Methods: A retrospective, descriptive, population-based epidemiological study using triangulation of aggregated secondary data from the Hospital Information System of the Unified Health System (SUS), the Statistical Yearbook of Social Security (AEPS)/National Social Security Institute (INSS), and the Institute of Applied Economic Research database (IPEADATA). Hospital Admission Authorizations related to microsurgical peripheral nerve grafts, microneurolysis, and microneuroplasty were included. Nominal hospital costs were adjusted to December 2024 using the Broad National Consumer Price Index.

Results: A total of 36,177 Hospital Authorizations were identified. Surgical production increased from 2,849 procedures in 2014 to 4,367 in 2019, decreased by 50.2% in 2020, and reached 3,639 procedures in 2024, remaining below the pre-pandemic level. Cumulative hospital expenditure totaled R$37.47 million in nominal values and R$49.69 million after inflation adjustment. In a separate and broader aggregated social security dataset, the accumulated initial monthly income associated with traumatic hand, wrist, and related upper-limb injuries exceeded R$527.6 million. Because the social security population was not restricted to individuals undergoing microsurgical nerve repair and could not be linked to SIH/SUS records, this amount was interpreted exclusively as contextual ecological information rather than as a cost attributable to the microsurgical cohort. The predominant hospital profile was male (81.0%) and 20-39 years old (65.0%), with most admissions classified as urgent (78.0%).

Conclusion: Microsurgical peripheral nerve repair generated substantial hospital utilization and inflation-adjusted expenditure within the Brazilian Unified Health System. Broader social security data additionally demonstrate the considerable economic magnitude of disability related to traumatic hand, wrist, and upper-limb injuries; however, these aggregated costs cannot be attributed specifically to patients undergoing microsurgical nerve repair. Timely access to reconstruction, integrated rehabilitation, and improved occupational surveillance remain relevant public health priorities.

## Introduction

The human hand is a highly specialized functional structure whose performance depends on the integrity of the peripheral nervous system for fine motor coordination, protective sensation, object manipulation, and interaction with the environment. Peripheral nerve injuries can cause persistent sensory, motor, and neuropathic pain deficits, with substantial consequences for independence, occupational performance, and return to work. The classic classifications proposed by Seddon and Sunderland remain essential for understanding the severity of nerve injury, as they relate axonal rupture and connective tissue involvement to prognosis, regenerative potential, and the need for surgical intervention [1,2].

When nerve continuity is interrupted, microsurgical repair becomes a key determinant of functional recovery. The principles of tension-free repair, interfascicular nerve grafting, and preservation or maintenance of neural architecture support the indication for neuroraphia, neurolysis, or nerve grafting, depending on the extent and pattern of the injury [3,4]. Contemporary references in hand surgery also emphasize that nerve repair must be understood within a broader reconstructive framework that includes soft tissue management, sensory recovery, rehabilitation, and functional reintegration [5].

In Brazil, peripheral nerve injuries frequently affect adults of working age and are commonly associated with traumatic mechanisms involving the upper extremity. National and international epidemiological studies have shown that these injuries frequently occur in economically active populations and result in specific, long-term occupational consequences [6,7]. Therefore, peripheral nerve injury is not only a surgical condition but also a public health issue with implications for work capacity, rehabilitation, and social protection.

The economic burden of peripheral nerve injuries extends beyond initial hospitalization. Previous cost analyses have revealed that treatment, rehabilitation, productivity losses, prolonged work absences, and disability-related costs can significantly exceed direct hospital expenses [8,9]. This distinction is particularly relevant in publicly funded healthcare systems, where the cost of urgent surgical care and the cost of long-term disability may be distributed across different public sectors.

In the Brazilian context, the Hospital Information System of the Unified Health System (SUS) allows for the assessment of hospital output and approved hospital expenditures, while social security data provide information on disability-related benefits. The Institute of Applied Economic Research database (IPEADATA) also allows for inflation adjustment using historical economic time series, enabling the comparison of expenditures from different years in real terms [10-12]. However, these databases are aggregated and do not allow for a direct link between each hospital procedure and each social security benefit.

An isolated assessment of hospital expenditures may therefore underestimate the broader economic impact of microsurgical repair of peripheral nerves. This study aimed to analyze the epidemiological profile and inflation-adjusted hospital expenditure associated with microsurgical peripheral nerve repairs performed in Brazil from 2014 to 2024 and, separately, to contextualize these findings through an ecological analysis of aggregated social security indicators related to traumatic hand, wrist, and related upper-limb injuries, without patient-level linkage between the hospital and social security databases.

## Materials and methods

This was a retrospective, descriptive epidemiological study with an aggregated economic analysis. The report was organized according to principles applicable to observational studies using aggregated secondary public data, including the Strengthening the Reporting of Observational Studies in Epidemiology (STROBE) reporting recommendations when relevant [13].

Data collection covered the period from January 2014 to December 2024. Three public data sources were used: the Hospital Information System of the Brazilian Unified Health System (SIH/SUS), accessed through the Tabulation Program for the DATASUS Information System (TABNET)/Department of Informatics of the Unified Health System (DATASUS), for hospital production and approved hospital expenditure; the Statistical Yearbook of Social Security (AEPS)/National Social Security Institute (INSS) for aggregated disability and occupational accident data; and IPEADATA for the historical series of the Extended National Consumer Price Index (IPCA) [10-12]. The extraction routes, filters, units of analysis, variables, and operational criteria for each data source are detailed in Table 1.

**Table 1:** Data sources and operational criteria SIH/SUS: Hospital Information System of the Brazilian Unified Health System; TABNET: Tabulation Program for the DATASUS Information System; DATASUS: Department of Informatics of the Unified Health System; SIGTAP: Unified Health System Management System of Procedures, Medications, Orthotics, Prosthetics, and Special Materials; AIHs: Hospital Admission Authorizations; AEPS: Social Security Statistical Yearbook; INSS: National Social Security Institute; ICD-10: International Statistical Classification of Diseases and Related Health Problems, Tenth Revision; IPEADATA: Institute of Applied Economic Research database; IPCA: Broad National Consumer Price Index

Component	Data source and query route	Period and filters	Variables extracted	Unit of analysis and analytical treatment
Hospital production and expenditure	SIH/SUS → TABNET/DATASUS → Health Care → Hospital Production (SIH/SUS) [10]	Brazil; January 2014-December 2024; SIGTAP codes 0403020018, 0403020026, 0403020050, and 0403020069	Approved AIHs, approved nominal hospital value, year, macroregion, demographic characteristics when available, and admission type	AIH. No patient-level deduplication; one individual may contribute more than one AIH
Social security contextual component	AEPS/INSS disability benefit and occupational accident statistics [11]; 2014-2024; traumatic hand, wrist, and related upper-limb injury categories, with emphasis on ICD-10 S64 when specifically available; aggregated benefit records only; no individual-level linkage with SIH/SUS.	2014-2024; traumatic hand/wrist and related upper-limb injury categories; emphasis on ICD-10 S64 when specifically available	Number and type of benefits, accumulated initial monthly income, disability pensions, and occupational accident notification when available	Aggregated benefit records. Broader population than the SIH/SUS procedure-specific component; no individual linkage
Economic adjustment	IPEADATA → monthly IPCA general index series [12]	Hospital expenditure from 2014-2024; reference date December 2024	Monthly IPCA index and accumulated correction factors	Nominal hospital expenditure converted to Dec 2024 Brazilian reais
Missing/unavailable information	Original SIH/SUS and social security classifications [10,11]	Variables available at the corresponding level of public aggregation	Categories reported by the original databases	No statistical imputation or patient-level reconstruction was performed. Analyses were restricted to available aggregated information. When complete denominators or category distributions were unavailable, they were not inferred.
Repeated hospital records	SIH/SUS [10]	All eligible AIHs	Approved hospitalizations/procedures	AIH rather than unique patient; repeated eligible AIHs from the same individual may be counted separately
Cross-database interpretation	SIH/SUS and AEPS/INSS [10,11]	Entire study period	Hospital expenditure and broader social security indicators	Contextual ecological comparison only; no patient-level linkage, causal attribution, or direct cost ratio
Extraction dates	DATASUS, AEPS/INSS, and IPEADATA [10-12]	DATASUS: Apr 23, 2026; AEPS/INSS: April 10, 2026; IPEADATA: April 23, 2026	-	Dates correspond to the final database consultation/extraction used in the analysis

For the hospital component, data were accessed through the following route: DATASUS → Health Information (TABNET) → Health Care → Hospital Production (SIH/SUS) [10]. The query was restricted to Brazil, from January 2014 through December 2024, and included Hospital Authorizations (AIHs) associated with the following Unified Health System Management System of Procedures, Medications, Orthotics, Prosthetics, and Special Materials (SIGTAP) procedure codes: 0403020018, microsurgical graft of two or more peripheral nerves; 0403020026, microsurgical graft of a single peripheral nerve; 0403020050, peripheral nerve microneurolysis; and 0403020069, microneurorrhaphy. The filters and variables used in the hospital extraction are summarized in Table 1. SIH/SUS data were accessed on April 23, 2026 [10].

Hospital variables included year, number of approved AIHs, approved nominal hospital value, macroregion, demographic characteristics when available, and type of admission. The unit of analysis was the AIH rather than the individual patient. Because the publicly available aggregated TABNET output does not provide patient-level identifiers suitable for deterministic linkage or deduplication, repeated hospitalizations or eligible procedures involving the same individual may generate more than one AIH and may therefore be counted more than once during the study period. Consequently, the number of AIHs represents hospital utilization and approved hospitalizations or procedures rather than the number of unique patients (Table 1).

For the social security component, aggregated information was obtained from the AEPS and related occupational accident statistics [11], particularly the sections addressing disability benefits and occupational accidents. The extraction considered accident-related disability benefits and disability pensions associated with traumatic injuries of the hand, wrist, and related upper-limb categories within the level of diagnostic detail available in the source databases, with emphasis on the International Statistical Classification of Diseases and Related Health Problems, Tenth Revision (ICD-10) code S64 (injury of nerves at wrist and hand level) when specifically available. Social security variables included the number and type of benefits, accumulated initial monthly income, disability pensions related to occupational accidents, and occupational accident notification information when available. These data were analyzed exclusively in aggregated form and were not linked at the individual level to SIH/SUS hospital records.

Importantly, the social security component was not restricted to individuals who underwent the microsurgical procedures identified in SIH/SUS. The diagnostic and administrative scope of the social security data was broader than the procedure-specific hospital cohort, and individual-level linkage between the two databases was not possible. Therefore, the social security data were used exclusively as a contextual ecological indicator of the broader economic magnitude of work disability associated with traumatic hand, wrist, and related upper-limb injuries. They were not interpreted as social security costs attributable specifically to patients undergoing microsurgical peripheral nerve repair, and no patient-level or causal economic comparison between the two populations was performed (Table 1).

For the economic adjustment of hospital expenditure, the monthly IPCA general index series was obtained from IPEADATA [12]. Nominal annual hospital values were converted to December 2024 Brazilian reais using the corresponding accumulated IPCA correction factors. The adjustment factor was applied to the approved hospital value for each year before calculation of the inflation-adjusted cumulative hospital expenditure. IPEADATA was accessed on April 23, 2026 [12].

Data were analyzed using descriptive statistics, including absolute frequencies, relative frequencies, annual trends, and financial totals. For variables unavailable or unspecified in the original aggregated databases, no patient-level reconstruction or statistical imputation was performed; analyses involving these characteristics were restricted to the information available in the corresponding database. No individual records were reconstructed, and no linkage was performed across SIH/SUS, AEPS/INSS, or IPEADATA (Table 1). For the inflation adjustment, only hospital expenditure was converted to constant December 2024 Brazilian reais. For each year of the study period, the nominal approved hospital expenditure was multiplied by the corresponding cumulative IPCA correction factor between the reference period and December 2024, obtained from the IPEADATA historical series [12]. The resulting annual inflation-adjusted values were subsequently summed to calculate cumulative hospital expenditure in December 2024 prices. Social security monetary values were not subjected to inflation adjustment, extrapolation, or patient-level estimation. The accumulated initial monthly income was retained as reported in the aggregated AEPS/INSS source [11] and was used exclusively as a contextual ecological indicator. Following the revised analytical framework, hospital and social security monetary values were neither summed nor divided to generate a cost ratio because the underlying populations are not equivalent and cannot be linked at the individual level. Missing, unavailable, or unspecified information in the original aggregated databases was not statistically imputed or reconstructed. Analyses were restricted to the information available for each variable in the corresponding source database. When a complete denominator or disaggregated category distribution was not retained in the extracted dataset, no numerator, residual category, or denominator was inferred from other variables [10-12].

No patient-level clinical scores, functional scales, or patient-reported outcome measures were applied or calculated in this study. The analysis was based exclusively on administrative and economic classifications available in aggregated public databases. The operational classification systems included SIGTAP procedure codes for hospital production, ICD-10 code S64 when available in social security records, AIH as the hospital unit of analysis, admission type recorded in SIH/SUS, occupational accident notification status when available, and IPCA as the inflation adjustment index [10-12].

Because this study used secondary, aggregated, publicly available data without any possibility of individual identification, submission to the Brazilian Research Ethics Committee/National Research Ethics Commission system was not required under Resolution No. 510/2016 of the Brazilian National Health Council [14].

## Results

**Figure 1:**
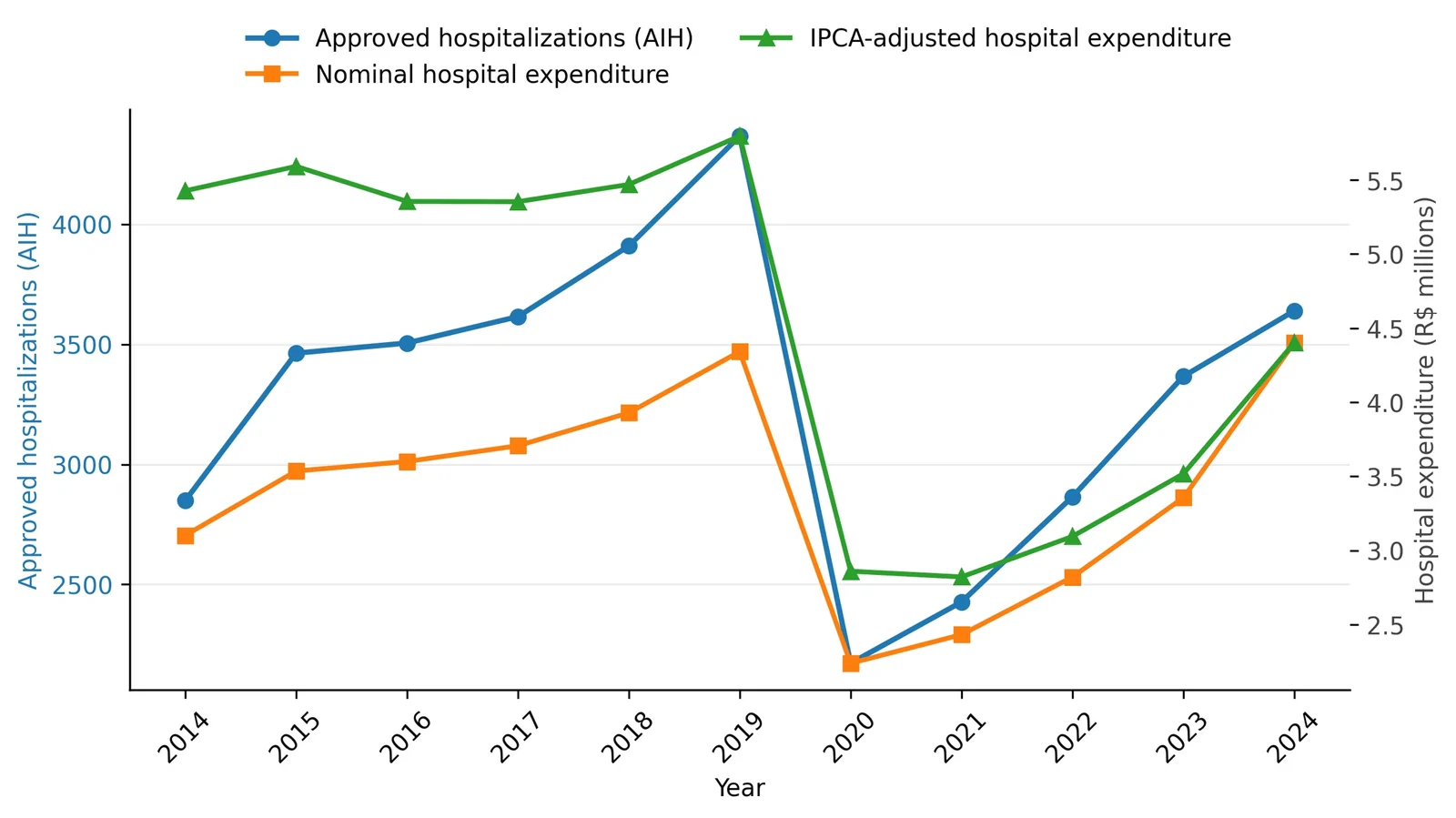
Annual trends in approved Hospital Authorizations and hospital expenditure for microsurgical peripheral nerve repair in Brazil, 2014-2024 IPCA: Extended National Consumer Price Index The figure was created by the authors using aggregated SIH/SUS/DATASUS hospital production data and IPCA-adjusted values calculated from IPEADATA. Values are expressed in Brazilian reais and adjusted to December 2024 when indicated.

Between 2014 and 2024, 36,177 AIHs were approved for peripheral nerve repair procedures in the SUS. The number of surgeries performed increased from 2,849 procedures in 2014 to 4,367 in 2019. In 2020, the number of procedures decreased to 2,172 AIHs, representing a 50.2% reduction compared to 2019. Starting in 2021, a gradual recovery was observed, reaching 3,639 procedures in 2024, though still below pre-pandemic levels. The annual trends in approved AIHs and hospital expenditure are shown in Figure 1.

Cumulative hospital expenses totaled R$ 37,470,321.17 in nominal terms. After adjusting for the IPCA for December 2024, the cumulative real value was R$ 49,687,616.40. In 2014, the nominal expenditure of R$ 3,102,496.39 corresponded to R$ 5,426,266.19 in 2024 values. In 2024, the approved amount was R$ 4,401,125.05, representing a real reduction of 18.9% compared to the 2014 amount adjusted for inflation. The annual hospital production and corresponding nominal and IPCA-adjusted expenditure are detailed in Table 2.

**Table 2:** Hospital production and approved values for microsurgical peripheral nerve repair in the Brazilian Unified Health System, 2014-2024 AIH: Hospital Authorization; IPCA: Extended National Consumer Price Index

Year	Approved AIH	Nominal value (R$)	IPCA-adjusted value to Dec/2024 (R$)
2014	2,849	R$31,02,496.39	R$54,26,266.19
2015	3,463	R$35,36,536.06	R$55,91,263.51
2016	3,505	R$36,00,867.43	R$53,54,490.00
2017	3,615	R$37,07,068.55	R$53,53,007.00
2018	3,909	R$39,28,912.18	R$54,69,045.75
2019	4,367	R$43,41,524.41	R$57,95,935.09
2020	2,172	R$22,40,341.42	R$28,60,916.00
2021	2,427	R$24,33,063.67	R$28,22,353.86
2022	2,865	R$28,21,743.24	R$30,95,452.33
2023	3,366	R$33,56,642.77	R$35,17,761.62
2024	3,639	R$44,01,125.05	R$44,01,125.05
Total	36,177	R$3,74,70,321.17	R$4,96,87,616.40

The cumulative nominal total was calculated by summing the annual nominal values presented in Table 2. In the social security component, the aggregate analysis identified 336,766 disability benefits resulting from work-related accidents during the reference period and 2,437 disability pensions related to work-related accidents involving the upper limb, within the scope of the database used. The cumulative initial monthly income exceeded R$ 527.6 million, with the highest annual value observed in 2024, when it exceeded R$ 67.7 million. These social security records represent a broader spectrum of traumatic injuries to the hand, wrist, and upper limbs than the SIH/SUS microsurgical dataset specific to the procedure. Consequently, these figures are presented solely as contextual ecological indicators and should not be interpreted as social security expenditures attributable to individuals who underwent microsurgical repair of peripheral nerves. The hospital and social security economic components and their respective scopes are summarized in Table 3.

**Table 3:** Aggregated comparison between hospital expenditure and social security burden during the study period SIH/SUS: Hospital Information System of the Brazilian Unified Health System; AIH: Hospital Authorization; IPCA: Extended National Consumer Price Index

Component	Population/scope	Economic measure	Accumulated value	Interpretation
SIH/SUS hospital component	AIHs for selected microsurgical peripheral nerve procedures	Approved hospital expenditure	R$37.47 million nominal; R$49.69 million IPCA-adjusted	Direct hospital expenditure within the procedure-specific study scope
Social security contextual component	Broader traumatic hand, wrist, and related upper-limb injury categories	Accumulated initial monthly income of disability-related benefits	>R$527.6 million	Contextual ecological indicator; not restricted or attributable to microsurgical patients

This is an ecological comparison based on aggregated databases and does not represent the individual link between hospital procedures and social security benefits.

Epidemiological and care-related variables derived from SIH/SUS/DATASUS [10] showed a predominance of male records (81.0%). Among the age-group data included in the consolidated dataset, individuals aged 20 to 39 years represented the predominant category (65.0%). Since the complete, disaggregated age distribution, as well as the corresponding numerators and denominators for the other age groups, were not preserved in the consolidated dataset used in this analysis, the residual 35.0% was neither interpreted nor presented as a distinct epidemiological category. No attempt was made to reconstruct the unavailable age distribution.

The regional distribution, also obtained from the SIH/SUS/DATASUS [10], showed that the Southeast accounted for 48.0% of the records, followed by the Northeast (20.0%), South (15.0%), Midwest (10.0%), and North (7.0%). Regarding the type of hospitalization, 78.0% of the SIH/SUS records were classified as urgent and 22.0% as elective [10]. Reports of work-related accidents were obtained separately from the aggregated social security data from AEPS/INSS [11] and were identified in 28.0% of the records for which this information was available. Since the hospital and social security databases represent different aggregate populations, the percentage of reported work-related accidents should not be interpreted as referring specifically to the SIH/SUS microsurgery cohort.

**Figure 2:**
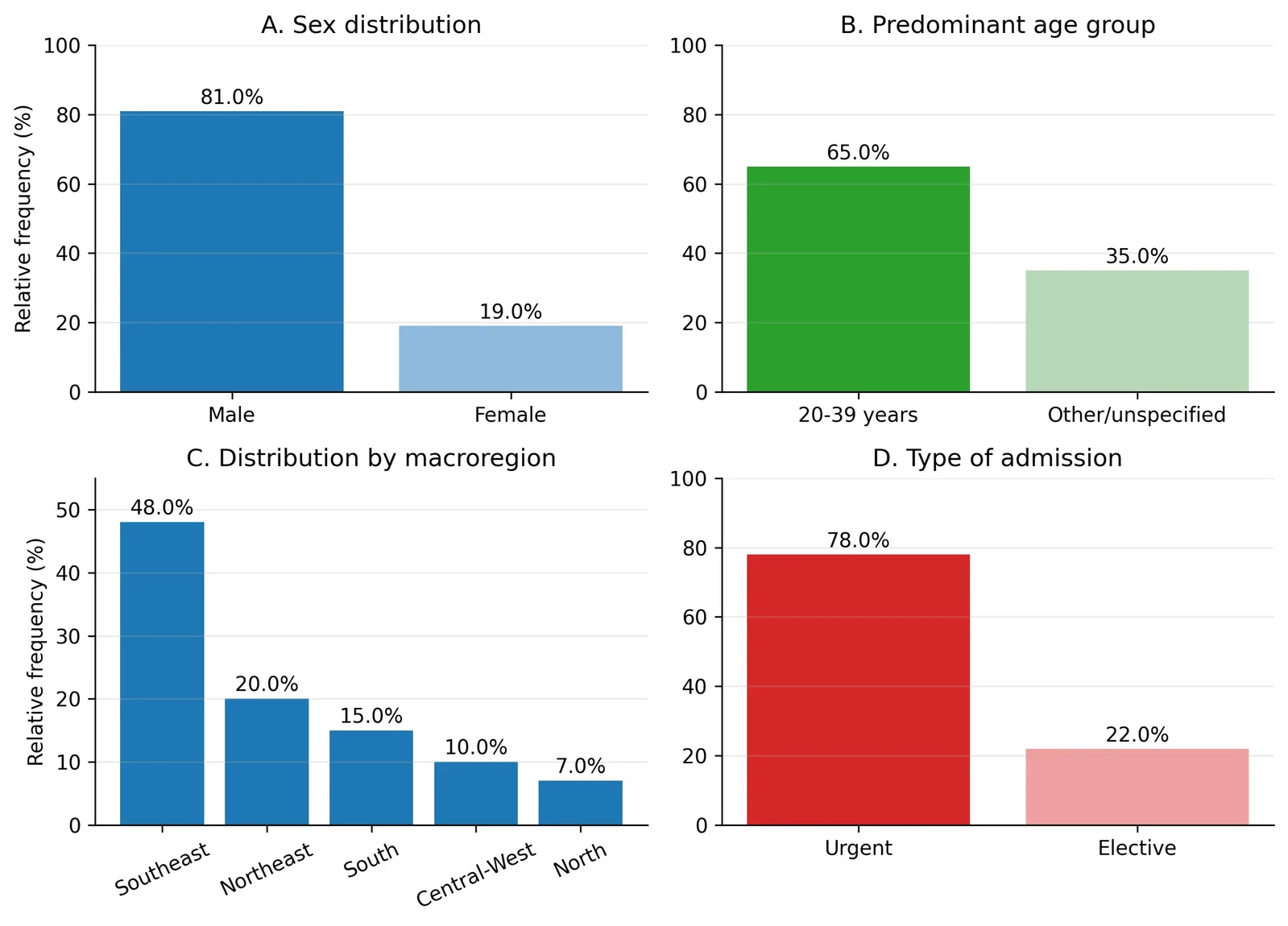
Epidemiological and care characteristics available from SIH/SUS/DATASUS, 2014-2024 Sex, macroregional distribution, and type of admission were obtained from SIH/SUS/DATASUS [10]. For age, only the proportion corresponding to the predominant age group (20-39 years; 65.0%) was retained in the consolidated extraction used in this analysis. Because the complete distribution across the remaining age categories and their corresponding numerators and denominators was not available, no residual “other/unspecified” age category is displayed. SIH/SUS: Hospital Information System of the Brazilian Unified Health System; DATASUS: Department of Informatics of the Unified Health System

Table 4 retains the original graphs showing distribution by sex, predominant age group, macroregional distribution, and type of admission. These epidemiological and care-related characteristics are summarized in Figure 2 and detailed in Table 4.

**Table 4:** Consolidated epidemiological and care profile of records analyzed, 2014-2024 * The consolidated extraction retained only the proportion corresponding to the predominant age group and did not include the complete disaggregated age distribution or the corresponding numerators and denominators. Therefore, the remaining 35.0% was neither classified as “other” nor treated as a separate age category. ^†^ Occupational accident notification was obtained from the aggregated AEPS/INSS dataset and does not refer specifically to the SIH/SUS microsurgical cohort. SIH/SUS: Hospital Information System of the Brazilian Unified Health System; DATASUS: Department of Informatics of the Unified Health System; AEPS: Social Security Statistical Yearbook; INSS: National Social Security Institute

Category	Variable	Relative frequency (%)	Data source
Demographic profile	Male sex	81.0	SIH/SUS-DATASUS [10]
Demographic profile	Female sex	19.0	SIH/SUS-DATASUS [10]
Age group	Predominant available age group: 20-39 years*	65.0	SIH/SUS-DATASUS [10]
Regional distribution	Southeast	48.0	SIH/SUS-DATASUS [10]
Regional distribution	Northeast	20.0	SIH/SUS-DATASUS [10]
Regional distribution	South	15.0	SIH/SUS-DATASUS [10]
Regional distribution	Central-West	10.0	SIH/SUS-DATASUS [10]
Regional distribution	North	7.0	SIH/SUS-DATASUS [10]
Type of admission	Urgent	78.0	SIH/SUS-DATASUS [10]
Type of admission	Elective	22.0	SIH/SUS-DATASUS [10]
Occupational notification	Occupational accident notification issued^†^	28.0	AEPS/INSS [11]

Comunicação de Acidente de Trabalho (CAT) refers to the Brazilian Work-Related Accident Report. The 28.0% occupational accident notification figure was derived from the aggregated AEPS/INSS records for which this information was available and does not refer specifically to the SIH/SUS microsurgical cohort. The remaining percentages reflect the available aggregated data in their respective source databases.

## Discussion

This study demonstrates a relevant volume of microsurgical peripheral nerve repair within the Brazilian Unified Health System and substantial inflation-adjusted hospital expenditure over the 11-year study period. The separate social security analysis also indicates that disability associated with the broader spectrum of traumatic hand, wrist, and related upper-limb injuries represents a substantial economic issue.

However, the two economic components address different populations and should not be interpreted as directly comparable estimates. Hospital expenditure was restricted to specific SIGTAP codes for microsurgical peripheral nerve procedures, whereas the social security data encompassed a broader set of traumatic injuries and disability-related benefits. Moreover, the aggregated nature of both databases precluded individual-level linkage between a hospital procedure and a subsequent social security benefit. The social security findings therefore provide contextual information regarding the broader economic environment in which peripheral nerve trauma occurs rather than an estimate of the indirect costs specifically generated by the microsurgical cohort.

This difference in monetary magnitude should be interpreted cautiously because the hospital and social security databases represent different aggregated populations and do not allow individual-level linkage between a surgical procedure and a subsequent benefit. Even so, the observed discrepancy is consistent with previous economic studies showing that peripheral nerve injuries generate costs that continue after the acute surgical episode. Rosberg et al. showed that median and ulnar nerve injuries in the forearm produced costs related not only to treatment but also to rehabilitation and lost production [8]. Bergmeister et al. also demonstrated that upper-extremity peripheral nerve injuries were associated with acute hospital costs, rehabilitation needs, sick leave, and long-term socioeconomic consequences [9]. These findings support the interpretation that hospital expenditure represents only a restricted portion of the full economic burden.

The abrupt reduction in procedures in 2020 probably reflects the systemic effect of the COVID-19 pandemic on surgical access, including the suspension or postponement of procedures in several services. In the context of peripheral nerve injuries, this finding is particularly relevant because nerve repair is time-sensitive. Delays may compromise axonal regeneration, increase the complexity of reconstruction, and reduce the likelihood of complete functional recovery [3-5]. Therefore, the 2020 reduction should not be interpreted only as a temporary reduction in hospital production, but also as a possible marker of interrupted access to specialized reconstructive care.

The predominance of young men of working age reinforces the occupational and socioeconomic character of the problem. This pattern is consistent with previous studies describing peripheral nerve injuries in economically active populations and linking upper-limb nerve trauma to functional impairment, rehabilitation needs, and difficulty returning to work [6-9]. In this sense, the consequences of peripheral nerve injuries may extend from the surgical field to the labor market and the social security system.

The low rate of formal occupational accident notification observed in this study suggests possible underreporting of the work-related causal link. This finding has important policy implications. Underreporting limits occupational surveillance, weakens prevention strategies, reduces the visibility of high-risk activities, and may impair structured return-to-work planning. Surgical treatment alone is unlikely to fully reduce the burden of these injuries if it is not integrated with rehabilitation, occupational health surveillance, and social security monitoring.

The real reduction in hospital financing between 2014 and 2024, despite nominal growth, suggests possible under-reimbursement of technically complex procedures. From a health policy perspective, reconstructive microsurgery should not be evaluated only by the immediate cost of hospitalization. It should also be assessed by its potential to reduce permanent disability, shorten work absence, decrease dependence on social security benefits, and improve long-term functional outcomes [8,9]. Early access to specialized microsurgical repair and integrated rehabilitation may therefore represent not only a clinical priority but also an economic sustainability strategy.

The main limitations arise from the use of secondary and aggregated administrative databases, which are subject to underreporting, coding inconsistencies, regional differences in data entry, and the inability to identify unique patients. The AIH represents an admission or procedure rather than an individual patient, and repeated eligible hospitalizations may therefore be represented more than once. In addition, the hospital and social security components represent different aggregated populations: hospital expenditure was restricted to selected microsurgical peripheral nerve procedures, whereas the social security data encompassed a broader spectrum of traumatic hand, wrist, and related upper-limb injuries. Because individual-level linkage between these databases was not possible, the social security findings should be interpreted exclusively as contextual ecological indicators and not as costs attributable to patients undergoing microsurgical nerve repair.

Accordingly, any numerical comparison between hospital and social security expenditures should be interpreted with particular caution. The substantially larger monetary magnitude observed in the social security dataset does not indicate that these costs were generated by, or are attributable to, the patients represented in the microsurgical SIH/SUS dataset. Rather, the social security component provides broader contextual information on the economic consequences of disability associated with traumatic hand, wrist, and related upper-limb injuries. The databases also do not provide patient-level clinical outcomes, functional recovery measures, rehabilitation trajectories, patient-reported outcomes, or complete productivity losses. Conversely, important strengths of the study include its national scope, the 11-year historical series, the use of standardized public administrative databases, and the inflation adjustment of hospital expenditure, which allows more meaningful temporal economic comparisons.

## Conclusions

From 2014 to 2024, microsurgical peripheral nerve repairs in the Brazilian Unified Health System accounted for 36,177 AIHs, with a marked decline in 2020 and only partial recovery in subsequent years. Inflation-adjusted hospital expenditure reached R$49.69 million over the study period. In a separate and broader aggregated social security dataset, accumulated initial monthly income associated with traumatic hand, wrist, and related upper-limb injuries exceeded R$527.6 million. However, because this population was not restricted to individuals undergoing microsurgical nerve repair and no patient-level linkage with SIH/SUS records was possible, this amount should be interpreted exclusively as a contextual ecological indicator and not as a social security cost attributable to the microsurgical cohort.

These findings support the relevance of timely access to specialized peripheral nerve treatment, functional rehabilitation, and occupational injury surveillance. Further studies using individually linked health and social security data are needed to quantify the direct and indirect economic consequences specifically attributable to peripheral nerve injuries and their surgical treatment.

## References

[1] Seddon HJ (1943). Three types of nerve injury. Brain.

[2] Sunderland S (1951). A classification of peripheral nerve injuries producing loss of function. Brain.

[3] Millesi H (1981). Interfascicular nerve grafting. Orthop Clin North Am.

[4] Lundborg G (2003). Richard P. Bunge memorial lecture. Nerve injury and repair - a challenge to the plastic brain. J Peripher Nerv Syst.

[5] Wolfe SW, Hotchkiss RN, Pederson WC, Kozin SH, Cohen MS (2017). Green’s Operative Hand Surgery. 7th Ed.

[6] Fuzari HKB, de Andrade AD, de Souza RJP, Bernardino SN, de Souza FHM, Sarmento A, de Oliveira DA (2021). Epidemiological profile of surgically-treated peripheral-nerve diseases. Arq Bras Neurocir.

[7] Kouyoumdjian JA (2006). Peripheral nerve injuries: a retrospective survey of 456 cases. Muscle Nerve.

[8] Rosberg HE, Carlsson KS, Höjgård S, Lindgren B, Lundborg G, Dahlin LB (2005). Injury to the human median and ulnar nerves in the forearm: analysis of costs for treatment and rehabilitation of 69 patients in southern Sweden. J Hand Surg Br.

[9] Bergmeister KD, Große-Hartlage L, Daeschler SC (2020). Acute and long-term costs of 268 peripheral nerve injuries in the upper extremity. PLoS One.

[10] (2026). Brazilian Ministry of Health: Hospital Information System of the Brazilian Unified Health System (SIH/SUS), DATASUS/TABNET. https://datasus.saude.gov.br/informacoes-de-saude-tabnet/.

[11] (2026). Statistical yearbook of social security - AEPS 2024. https://www.gov.br/previdencia/pt-br/assuntos/previdencia-social/arquivos/aeps-2024/aeps-2024.

[12] (2026). Institute for Applied Economic Research: IPEADATA historical series. https://www.ipeadata.gov.br/.

[13] von Elm E, Altman DG, Egger M, Pocock SJ, Gøtzsche PC, Vandenbroucke JP (2007). The Strengthening the Reporting of Observational Studies in Epidemiology (STROBE) statement: guidelines for reporting observational studies. Lancet.

[14] BRASIL BRASIL, GOV CNS (2026). Brazilian National Health Council: Resolution No. 510, April 7, 2016. Official Gazette of the Union, Brasília; 2016 [Website in Portuguese]. Brazilian National Health Council: Resolution No. 510, April 7.

